# The Transmissibility of the Human Skin Virome: Potential Forensic Implications

**DOI:** 10.1002/mbo3.70197

**Published:** 2025-12-08

**Authors:** Min‐Jeong Kim, Ji‐Ho Park, Yong‐Bin Eom

**Affiliations:** ^1^ Department of Medical Sciences, Graduate School Soonchunhyang University Asan Chungnam Republic of Korea; ^2^ Department of Biomedical Laboratory Science, College of Medical Sciences Soonchunhyang University Asan Chungnam Republic of Korea

**Keywords:** individual identification, skin virome, stability, transmissibility, viral marker

## Abstract

The objective of this study was to evaluate the temporal stability and object‐to‐skin transferability of the skin virome in a Korean population. Skin virus metagenomes were collected from the anatomical locations (forehead, left hand, and right hand) of eight healthy adults and monitored over 3 months at intervals of 6 weeks. To assess the potential transfer of virome between skin and objects, subjects were instructed to contact four types of objects (cell phones, door handles, fabric, and plastic). Virome samples were then collected from the surfaces of these objects. Viruses were identified using databases and viral annotation bioinformatics tools. Fifteen viral families were consistently found to be stable and well‐transmissible across anatomical locations and four types of objects. Furthermore, the presence/absence profiles of 54 viral species belonging to these 15 viral families exhibited significant individual specificity on both the skin (*p* < 0.01) and the objects handled by each subject (*p* < 0.05). We confirmed that these 54 viral markers remain stable over time within individuals and are transferable to contacted surfaces. Additionally, we explored the potential of using the virome as an individual identification marker, which may suggest new approaches for forensic applications.

## Graphical abstract

To robustly establish the forensic potential of the human skin virome, continuous research and investigation across diverse ethnic groups are necessary. This study evaluated the stability and transmissibility of the skin virome in Koreans. Our results show that the skin virome is stable over time and can be shared through contact with objects.

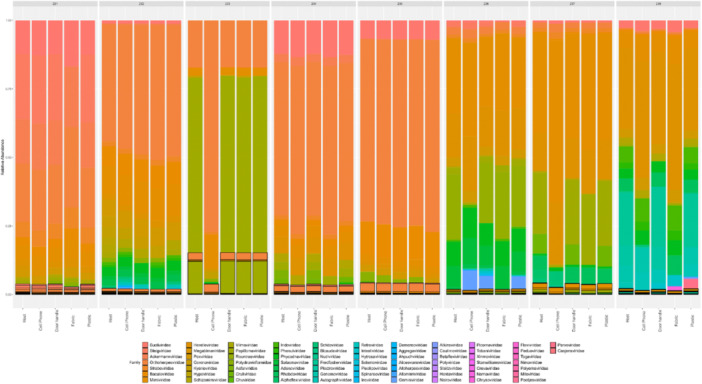

## 1 Introduction

Since its introduction in the 1980s, DNA has become a primary tool in forensic science (Jeffreys et al. 1985; Butler 2011, 2014). Moreover, with the advancement of Next‐Generation Sequencing (NGS) technology, the amount of genetic data available for forensic analysis has increased exponentially (Børsting and Morling 2015). As a result, the range of genetic material‐based evidence available for forensic investigation has expanded, providing valuable additional genetic information of significance as evidence (Kuiper 2016). Among this genetic information, the human microbiome is increasingly recognized for its value as a forensic tool (Clarke et al. 2017).

The human microbiome has been used as physical evidence for over a century (Perego et al. 2013). In the past, analysis technologies were generally slow and expensive, making it impractical to characterize various microbial communities, thus limiting their use in forensic science (Metcalf et al. 2017). However, recent massive parallel sequencing has made it possible to profile numerous DNA samples simultaneously based on low‐cost, high‐throughput technology (Alivisatos et al. 2015). This has significantly expanded the field of microbiology. Microbes associated with age, gender, lifestyle, and geographical location have gained potential in forensic science (Alarcón et al. 2016; López‐Goñi 2018; Cho and Eom 2021). Previous studies have demonstrated the potential to estimate post‐mortem interval using microbial community data (Pechal et al. 2014; Cobaugh et al. 2015; Hauther et al. 2015; Metcalf et al. 2016), identify individuals through skin microbes, or link human microbiome to objects and spaces (Meadow et al. 2014; Lax et al. 2015). In particular, one study has shown that skin bacteria could classify individuals with high accuracy even after 1 year (Watanabe et al. 2018).

However, despite many studies demonstrating the potential of microbial communities as forensic tools, there have been few studies using viruses closely associated with the microbiome for forensic purposes. Indeed, although it has been recognized that the diversity of microbial communities varies depending on human health and various environmental factors (Turnbaugh et al. 2007; Kostic et al. 2012; Haiser et al. 2013), it is significantly less recognized that the diversity of the human virome persists within an individual (Virgin et al. 2009; Virgin 2014; Kumata et al. 2020; Liang and Bushman 2021). According to one study, viruses can influence the structure and function of microbial communities, with the human skin virome having a dynamic association with microbial communities (Hannigan et al. 2015). Furthermore, several studies have confirmed the diversity of the virome, its stability over time, and its potential for use in individual identification (Shkoporov et al. 2019; Shah et al. 2023). Moreover, the human virome has been suggested to be useful in human identification and forensic applications (Graham et al. 2022). However, to robustly establish the forensic potential of human virome, additional research, and investigation across diverse racial groups are continually needed. Therefore, based on the hypothesis that viral markers could be used for human identification, the objective of this study was to determine the stability of skin virome over time in Koreans and further evaluate the possibility of linking skin virome with objects.

## 2 Experimental Procedures

### 2.1 Sample Collection

We recruited eight subjects (male‐to‐female ratio = 1:1) according to procedures approved by the Institutional Review Board (IRB) of Soonchunhyang University (IRB approval number: 1040875‐202306‐BR‐068). Subjects completed a questionnaire to assess intrinsic and extrinsic factors that might influence the skin virome profile on each sampling date (Figure S1). Based on the questionnaire responses, only subjects who met the study's inclusion criteria were included: (1) overall good health without observable skin disease; (2) age between 20 and 70 years; (3) no antibiotic use within the preceding 30 days; (4) no showering or handwashing for at least 2 h prior to sampling; and (5) ability to understand and provide informed consent for all study procedures. Virome samples were collected from three anatomical locations (forehead, left hand, and right hand) using sterile 4N6FLOQSwabs™ (Thermo Fisher Scientific) following the double‐swab technique described previously (Pang and Cheung 2007). Briefly, the targeted surface was wiped horizontally and vertically with a swab soaked in sterile 1× phosphate‐buffered saline (PBS), followed by the same wiping pattern using a dry swab. To evaluate the stability of the virome over time, samples were collected at 6 and 12 weeks after the initial sampling (Day 0), resulting in a total of three samples from each subject. To assess whether skin viral communities could be transferred to items handled either routinely or briefly, additional samples were collected from personal items (mobile phones and door handles) and from pre‐sterilized materials, including 100% cotton fabric and a plastic knife handle. For objects touched only briefly, subjects were instructed to rub or hold the surface with both hands for 1 min. Negative control swabs were collected to monitor potential contamination from sources such as PBS, swabs, and other experimental tools. Immediately after collection, all samples were transported to the laboratory on ice and stored at −80°C for up to 14 days before further processing.

### 2.2 Virome Extraction and Purification

The extraction and purification of viruses followed the procedure outlined previously (Graham et al. 2022). Swabs that collected virome from skin and objects were placed in sterile 2 mL microcentrifuge tubes containing a CW Spin Basket (Promega). Then, 200 μL of sterilized 1× PBS was dispensed into it. The PBS solution and virus particles were separated from swabs by centrifugation at 16,000 × *g* for 10 min. To eliminate cellular and bacterial contamination, the filtrate containing viral particles was further filtered using a 0.2 μm sterile membrane filter. Subsequently, viral nucleic acids were extracted using a QIAamp UltraSens Virus Kit (Qiagen) following the manufacturer's instructions. Viral nucleic acids underwent whole‐genome amplification using a 4BB™ TruePrime® Whole Genome Amplification (WGA) Kit (4basebio) according to the manufacturer's instructions. It used a multiple displacement amplification (MDA) method based on a combination of Phi29 polymerase. The concentration and purity of each sample were then assessed using a spectrophotometer (Infinium F‐200, NanoDrop, Illumina). DNA integrity was confirmed using an Automated Electrophoresis System (4200 TapeStation System, Agilent Technologies).

### 2.3 Library Preparation, Sequencing, and Assembly

Approximately 100 ng of amplified product was fragmented using an S220 Focused‐ultrasonicator (Covaris) for a mean length dispersal of 600 bp. Sequencing libraries were constructed using the NEBNext® Ultra™ II DNA Library Prep Kit (New England Biolabs) according to the manufacturer's instructions. After the process of end repair, A‐tailing, and adapter ligation, libraries were amplified with indexed primers. The quality of libraries was confirmed with a 4200 TapeStation System (Agilent Technologies) and a D5000 ScreenTape System (Agilent Technologies). Additionally, libraries were quantified using a KAPA Library Quantification Kit (Kapa Biosystems) according to the manufacturer's library quantification protocol. Following cluster amplification of denatured templates, paired‐end (150 bp) sequencing was performed on an Illumina Novaseq. 6000 System (Illumina). Raw sequencing reads were deposited in the NCBI Short Read Archive (SRA) under accession number PRJNA1073697. The quality of raw sequences was assessed using FastQC (v.0.11.9) (Andrews 2010) and filtered using Sickle (v.1.3.3) (Koparde et al. 2017). Briefly, a quality trimming threshold of Q30 was applied. Nucleotides less than 75 bp in length were removed from the dataset. Reads resulting from PhiX spike‐in were removed from cleaned reads using the BBDuk command (Bushnell 2014). Subsequently, bacterial contamination was assessed by mapping trimmed reads to the Silva 16S ribosome database (v.138.1) (Bushnell 2014). Additionally, to mitigate human host contamination, reads that were mapped to the human genome (hg19) were removed (Bushnell 2014). Metagenomic reads from samples were assembled with MEGAHIT (v.1.2.8) (Li et al. 2015). QUAST (v.5.0.2) (Gurevich et al. 2013) was used to assess assembly quality. Resulting virome assemblies were mapped to negative control contigs larger than 500 bp using BWA‐mem (Li 2013) to remove reads that might arise from potential contamination. Subsequent contigs larger than 500 bp were utilized for analysis to assess virus identification, diversity, stability, and transmissibility.

### 2.4 Viral Identification and Taxonomy

The quality of contigs longer than 500 bp after assembly was analyzed using CheckV (v.0.7.0) (Nayfach et al. 2021). Contigs identified as viruses by CheckV were classified using nucleotide‐based classification tools Kraken2 (v.2.0.8‐beta) (Wood et al. 2019) and BLASTn (Altschul et al. 1990). Homology analysis of contigs was performed against a local copy of the NCBI nucleotide database using BLASTn with an e‐value cutoff of 10^−5^. This was done to reduce misclassification of viral contigs caused by the algorithm and reference database. Classification of results was based on the lowest e‐value or the highest confidence hits.

### 2.5 Estimation of Viral Abundance

Read mapping was performed using Bowtie 2 (v.2.3.5) (Langmead and Salzberg 2012) and SAMtools (v.1.9) (Li et al. 2009) to generate abundance tables for each virus contig associated with each sample. Traditional taxonomic unit count tables and merged tables for sample read presence/absence were further analyzed using the Phyloseq package in R (v.3.6.3) (R Core Team, R 2024). The relative abundance of the top 10 most abundant virus families among skin viruses observed throughout the study was determined. Furthermore, to determine virus transmissibility, the abundance of all viral families observed within each subject was summed, regardless of sampling location. We assessed the relative abundance of the top 10 most abundant viral families for each object in contact with these hosts.

### 2.6 Virome Diversity

To quantitatively compare the viral similarity (α‐diversity) of each sample, the Shannon diversity index (Shannon 1948) was calculated. Analysis of variance (ANOVA) was employed to assess whether the continuous diversity of participants varied across body sites and objects. For evaluating intergroup diversity (β‐diversity), principal coordinates analysis was performed using the Jaccard dissimilarity index. Permutational multivariate analysis of variance (PERMANOVA) was utilized to test the statistical significance of differences between subjects (Oksanen et al. 2022). The analysis was conducted using the R statistical package along with R‐related tools Phyloseq and Vegan (McMurdie and Holmes 2013; R Core Team, R 2024; Oksanen et al. 2022).

### 2.7 Evaluation of Virome Stability Over Time

Virus stability was assessed based on their presence over time across anatomical locations sampled within each subject (forehead, left hand, right hand). Viral families present at least two of the three time points at each sampling location within an individual were identified. Furthermore, we identified stable virus families confirmed by at least one participant across all three anatomical locations. These were identified as potential viral markers for human identification. Subsequently, to investigate viral markers for human identification, stable viruses at the species level were identified. A Jaccard dissimilarity matrix was generated for the identified markers, where distances closer to 0 meant that classes were identical (0 = samples were identical, 1 = samples were disjoint). The Jaccard distance was averaged between a subject's sample and all samples excluding that subject (between‐subject dissimilarity) and across all samples collected from an individual (within‐subject dissimilarity). To assess the stability of markers, a series of pairwise Wilcoxon rank sum tests was performed for the between‐subject and within‐subject dissimilarity distances.

### 2.8 Evaluation of Skin Virome Transferability

The transmissibility of a virus was assessed by comparing the virus identified from each subject throughout the study with the virus found on objects that the subjects came into contact with. The evaluation was based on the presence of reliably transmitted virus families in two or more of the four objects with which an individual came into contact. At this time, transmissible viruses confirmed from one or more subjects were identified as putative markers showing the possibility of a connection between the subject and the virome remaining on the surface of the object in contact. Subsequently, a Jaccard dissimilarity matrix was generated for the identified markers, where distances closer to 0 meant that classes were identical (0 = samples were identical, 1 = samples were disjoint). The Jaccard distance was averaged between the object touched by the subject and all samples excluding that subject (between‐subject dissimilarity) and the distance between the four objects the individual touched (within‐object dissimilarity). To assess the stability and transferability of markers, a series of pairwise Wilcoxon rank sum tests was performed for between‐subject dissimilarity and within‐subject dissimilarity distances.

## 3 Results

### 3.1 Virome Quantification and Assembly

Virome samples were collected from the skin and four types of objects with which the subjects came into contact with. After whole‐genome amplification using MDA, metagenomic shotgun sequencing was performed. Overall, a total of 206,631 contigs were observed irrespective of contig size, with 46,608 contigs confirmed to be larger than 500 bp. Only contigs larger than 500 bp were used for subsequent analysis. Contigs of viral origin were analyzed using CheckV based on viral sequence information. A total of 2400 non‐redundant contigs were finally generated.

### 3.2 Taxonomic Identification of Skin Virome

**FIGURE 1:**
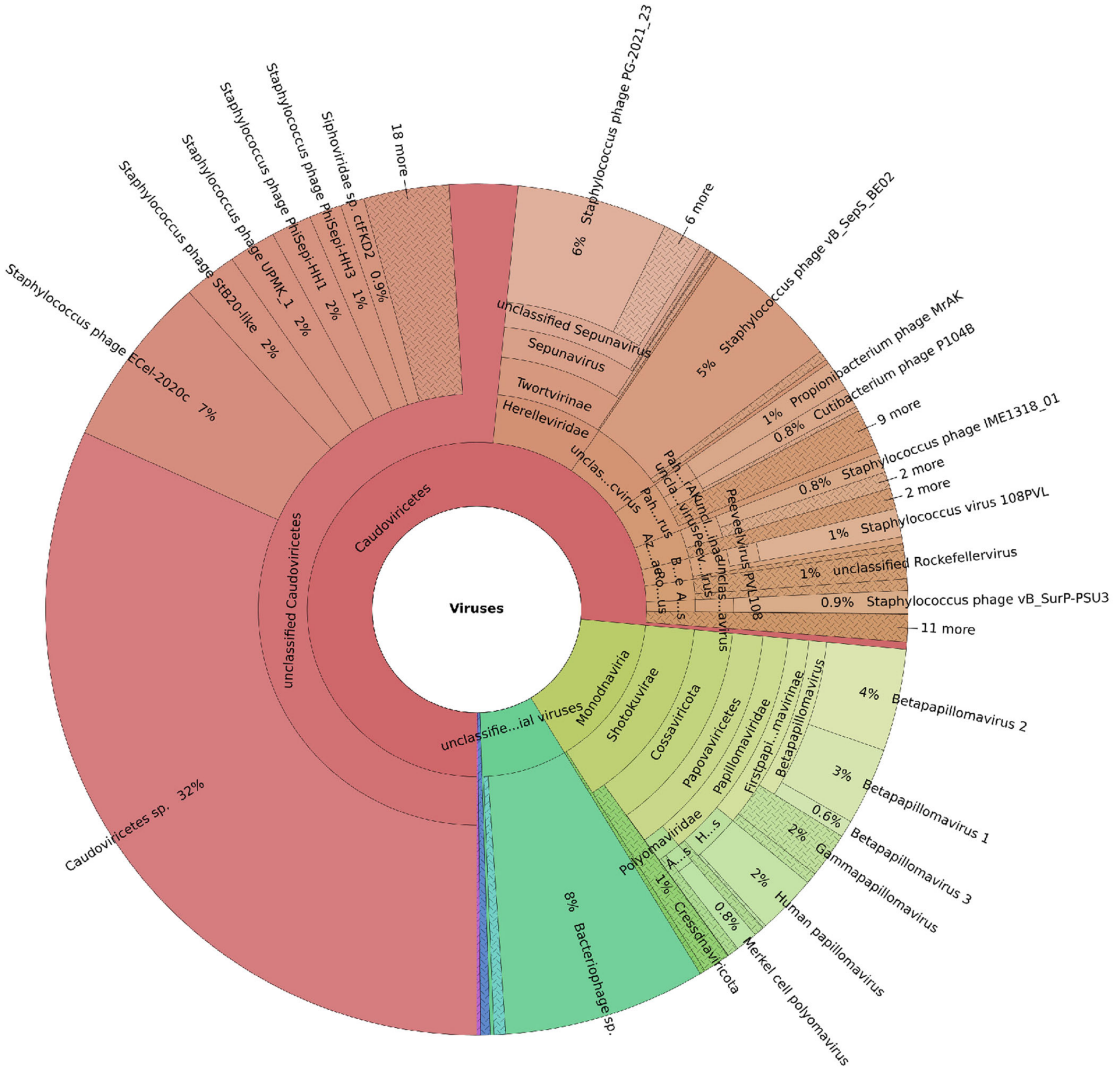
The distribution of viruses identified throughout the experiment in this study. Assembled contigs (>500 bp) of human skin metagenome samples identified by CheckV (v.0.7.0) were taxonomically identified using BLASTn. Unclassified viruses were excluded. The relative abundance of identified viruses was plotted in radial space.

All viruses identified in this study across the experiments, based on 2400 identified contigs using current virus databases and virus annotation bioinformatics tools, are shown in Figure 1. The majority of confirmed virus contigs could not be fully annotated using available virus reference databases or existing bioinformatics tools. Among identified DNA viruses, Caudoviricetes (76%) was the most abundant class, and Papovaviricetes (14%) was the second most abundant class.

### 3.3 Persistence and Transmissibility of the Virome

**FIGURE 2:**
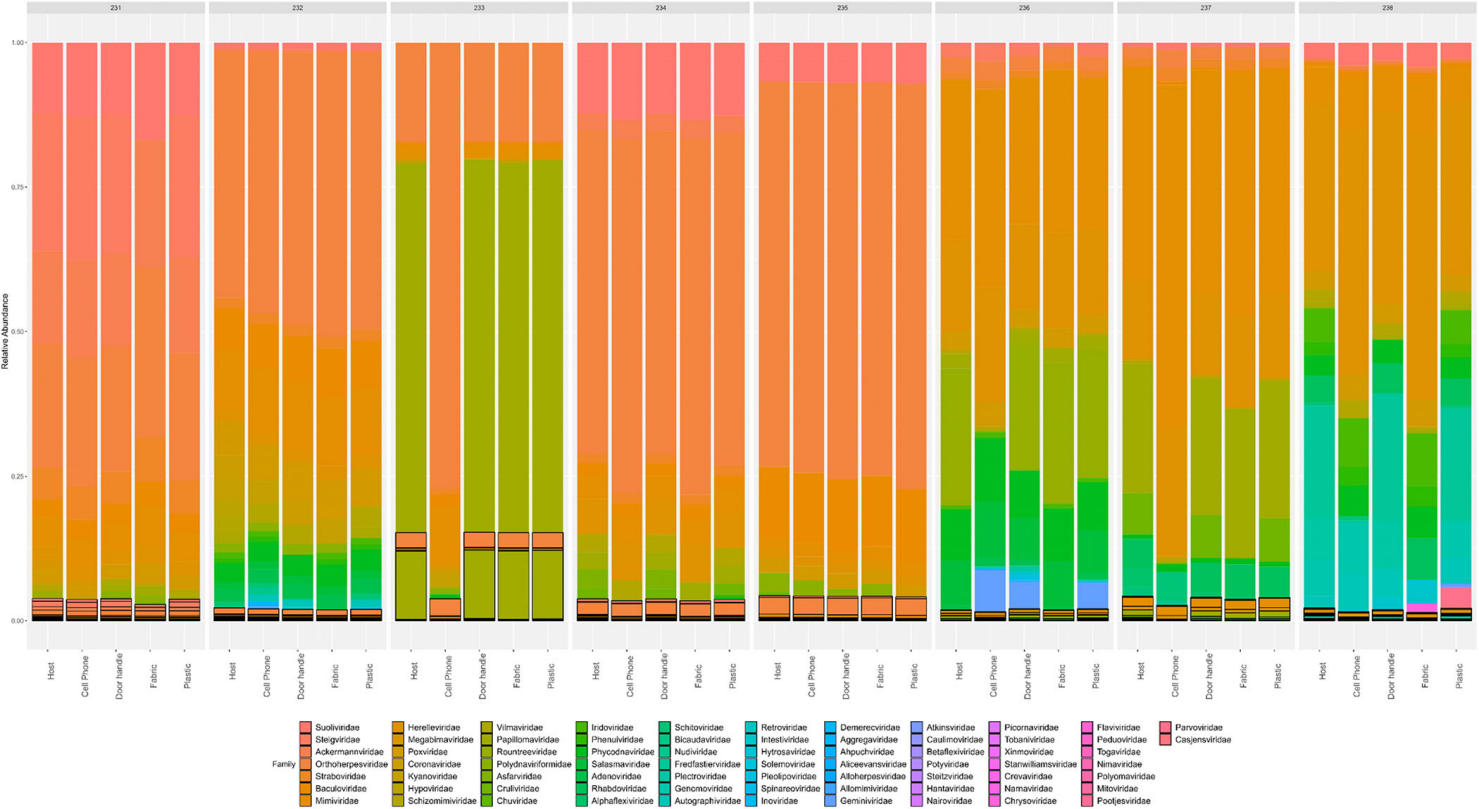
Relative abundance of the top 10 most abundant viruses identified on the subject's skin and contact objects. The subject's number is indicated at the top of the bar. For the “Host,” the relative abundance was calculated by summing across all sampling sites (forehead, left hand, and right hand) and all time points collected from the same subject. The following bars (Cell Phone, Door handle, Fabric, and Plastic) represent the relative abundance of viruses identified on the four types of objects that were in direct contact with the subjects. Contigs that could not be taxonomically classified at the family level using the current database were not included.

We sampled and monitored the skin virome of eight healthy adults every 6 weeks for 3 months. Three replicate observations were conducted per individual. We identified the top 10 most abundant virus families over time at three anatomical sites (forehead, left hand, and right hand) for each subject (Figure S2). Contigs that could not be taxonomically classified at the family level using the current database were excluded. The overall virome composition showed similarities between the anatomical locations of each subject. Furthermore, the relative abundance of the virome remained generally stable with little variation over time (Figure S2). We also identified the top 10 most abundant viral families in hosts (the subject's skin virome) and four types of objects (Figure 2). The abundance within hosts was summed across the subject's anatomical sampling locations and all time points. Among the most abundant viral families observed, a viral family of Caudoviricetes accounted for the majority. This was consistent with the skin virome diversity identified in all subjects (Figure 1). The virome abundance observed on the skin of each subject and the objects was similar. Particularly, despite differences in material and contact duration among the four types of objects, the relative abundance of the virome remained comparable (Figure 2). This suggests that viromes can be shared regardless of the material or contact time of the objects in contact with the skin (Figure 2).

### 3.4 Statistical Evaluation of Skin Virome

**FIGURE 3:**
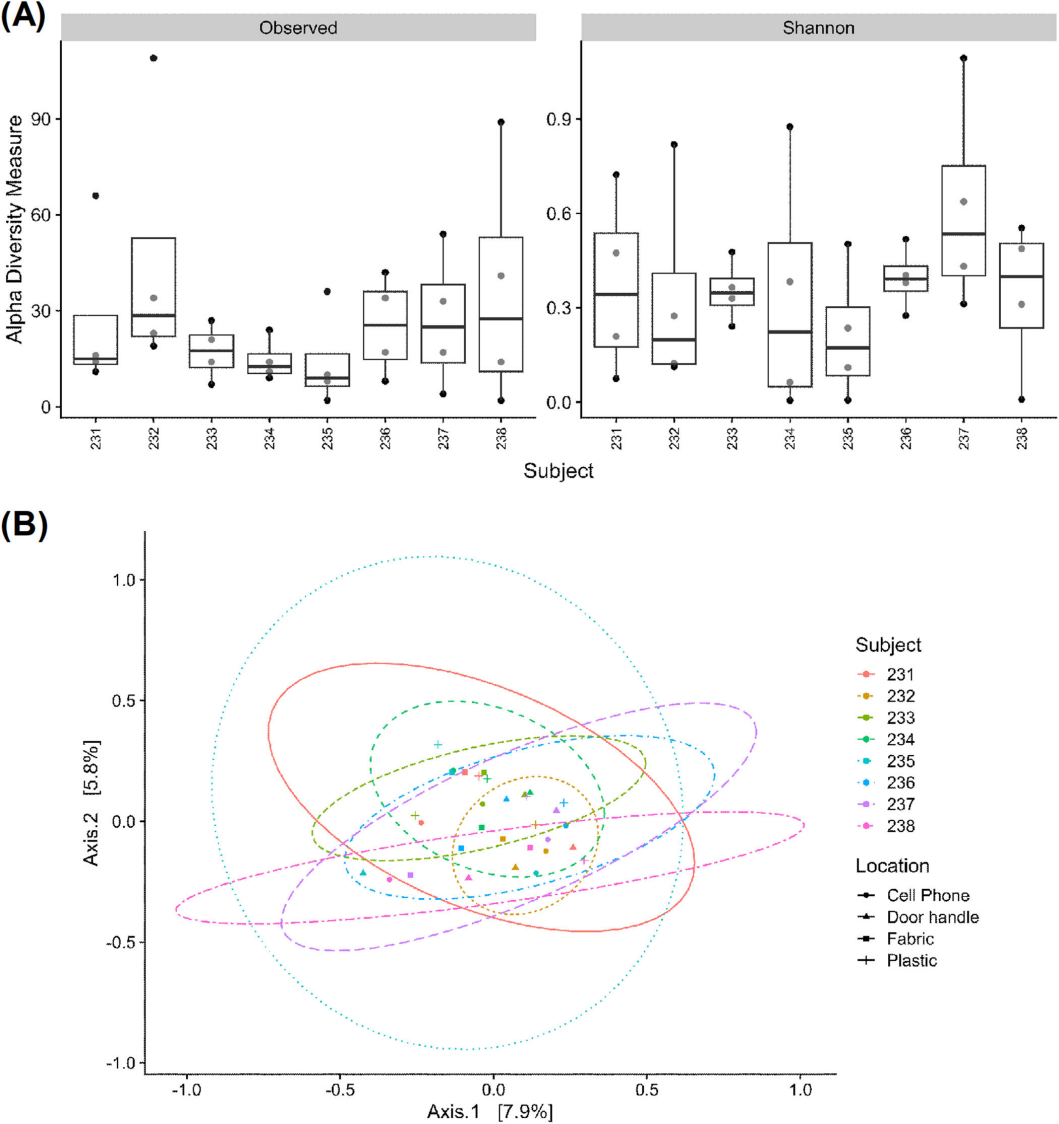
The diversity of the virome identified on objects in contact with skin. (A) Box plot comparing Shannon diversity measurement values for object samples contacted by subjects. ANOVA for the four types of objects derived from each subject showed no significance (*p* = 0.709). (B) PCoA plots of the Jaccard dissimilarity distance for β‐diversity assessment. In PERMANOVA, clustering by subject according to object virome showed significance (*R* ^*2*^ = 0.25, *p* = 0.012).

To determine the statistical significance of the diversity and distribution of these viruses, we conducted alpha and beta diversity analyses (Figure 3). We compared Shannon diversity measurements (α‐diversity index) for object samples contacted by subjects. ANOVA showed no significant differences across the four types of objects from each subject (Figure 3A, *p* = 0.709). This indicates that the distributions of shared viruses are consistently observed within an individual, regardless of the material or time of day. Inter‐subject diversity was performed via PERMANOVA using the Jaccard dissimilarity distance method. Inter‐subject specificity was observed in object virus clusters from eight subjects (Figure 3B, *p* = 0.012). Similarly, α‐diversity and β‐diversity were assessed for the skin samples of the subjects (Figure S3). This shows that the skin virome can be individualized and that the sharing of the virome between the skin and objects is differentiated for each subject.

### 3.5 Verification of Viral Markers for Personal Identification

**FIGURE 4:**
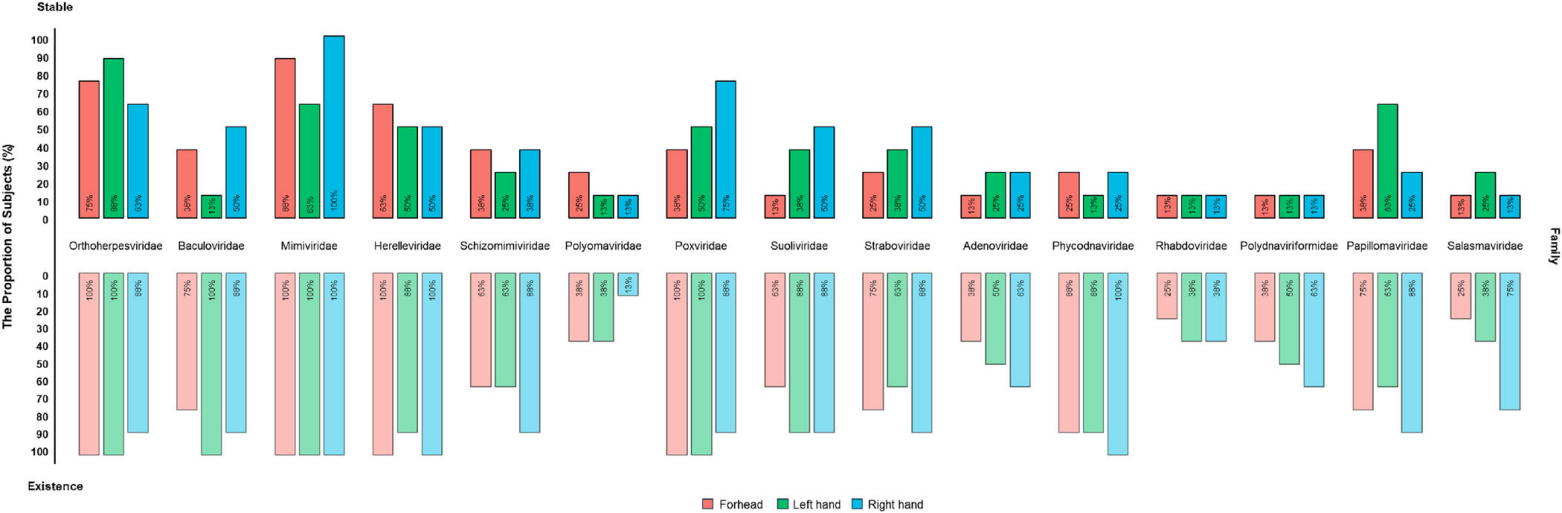
Stable virus families identified across anatomical locations of all subjects. Viruses detected at more than two of the three time points were considered stable. The proportion of subjects with stable viruses identified at anatomical locations (forehead, left hand, right hand) (top; Stable) and the proportion of subjects with viruses present at least once regardless of stability (bottom; Existence) are shown. Fifteen virus families, confirmed to be stable across all anatomical locations, were selected.

**FIGURE 5:**
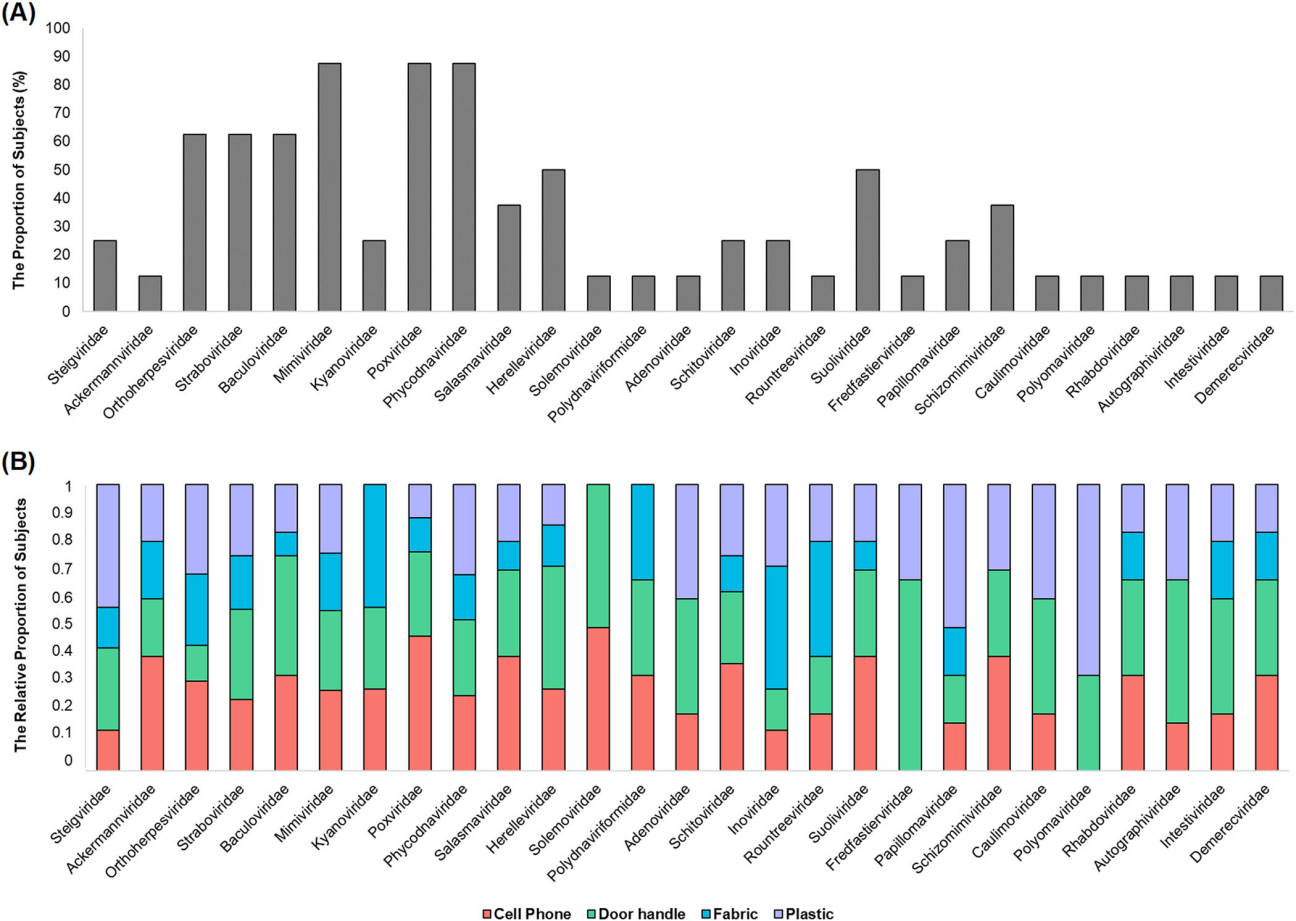
Virus families exhibiting stable transferability from skin to objects. Viruses found on two or more out of the four types of objects contacted by the subjects were considered stable. Twenty‐seven virus families, confirmed to be reliably transmitted, were selected. (A) A proportion of subjects where stably transferred viruses were observed. (B) Relative proportion observed in four objects for each virus family.

We characterized the virome by determining the stability, transmissibility, and individualization (Figures 2 and 3). Furthermore, we performed analyses of stable, transmissible, and individualized family‐level viruses to determine the potential use of the virome as a forensic marker for individual identification. Firstly, we identified virus families consistently observed over time at the anatomical location of subjects (Figure 4). Virus families observed at two or more time points out of the three sampling points were considered stable viruses. The proportion of subjects for whom stable virus families were identified at specific anatomical locations (top; stable) and the proportion of subjects for whom these viruses existed overall regardless of stability (bottom; existence) were determined. We identified 15 virus families consistently stable across all anatomical locations (Figure 4). Additionally, we identified virus families showing stable transmission from skin virome to objects (Figure 5). Viruses observed on two or more of four types of objects based on the subject's skin virome (Host) were considered to be stably transmitted. We found that 27 virus families were stably detected (Figure 5A). The proportion of participants with observed stable viruses is depicted in Figure 5A, indicating virus families transmitted stably to at least one individual. We plotted the ratio of transmission for each virus by object based on 27 virus families (Figure 5B). All viruses were observed to be transmitted to at least one individual via door handles. However, six virus families were not transmitted to the fabric.

### 3.6 Identification and Statistical Evaluation of Viral Markers

**FIGURE 6:**
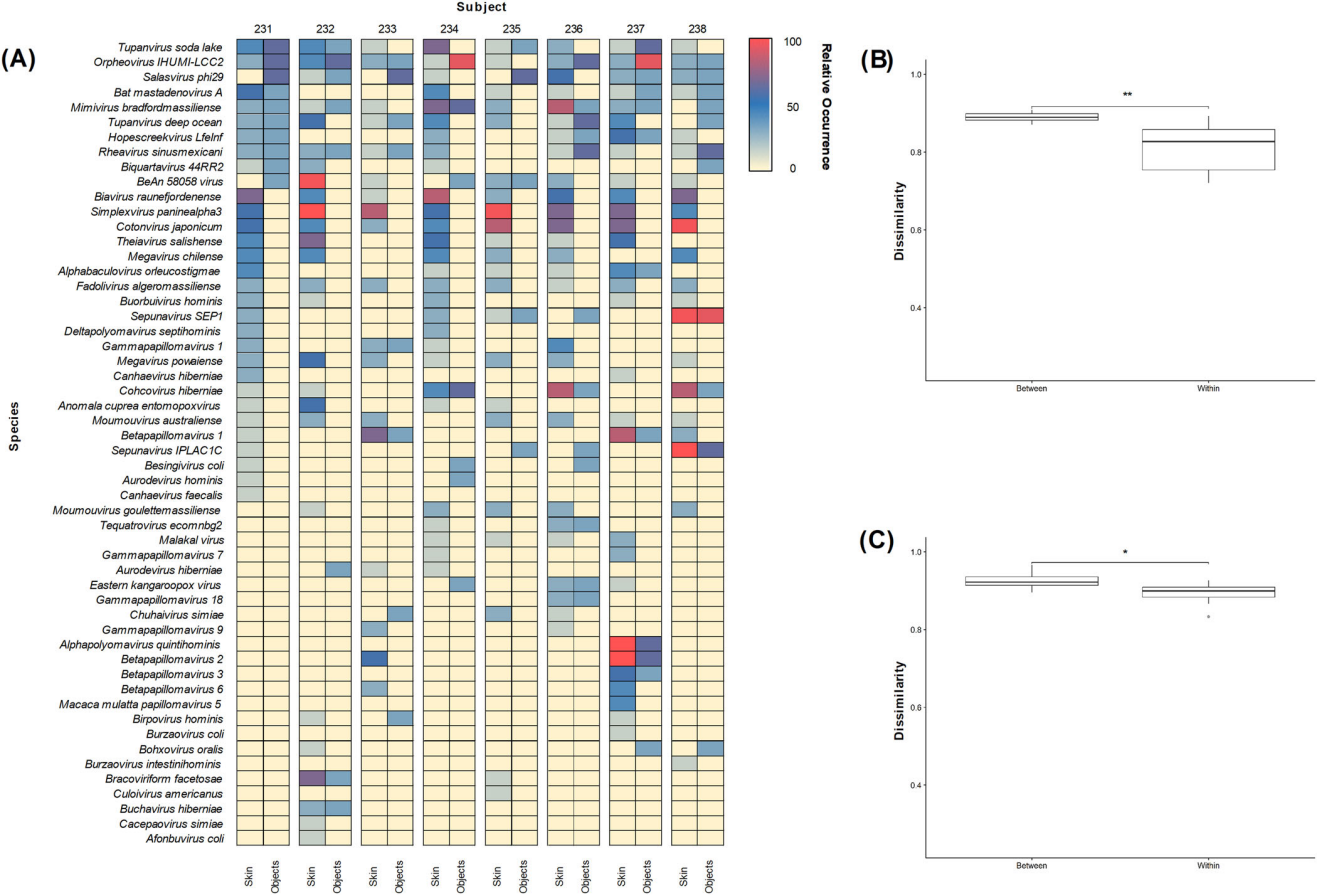
Fifty‐four viral species as viral markers. (A) Heatmap illustrates the stability and transferability of 54 selected viral markers. The label “Skin” represents the integration of anatomical locations (forehead, left hand, and right hand), while the label “Objects” combines four items (Cell Phone, Door handle, Fabric, and Plastic). Viral detection is expressed as relative occurrence (0–100%) across all sampling time points, where values closer to 100% indicate more consistent detection within an individual. A red–blue–yellow color scale was applied for visualization. (B, C) Statistical analysis of 54 virus markers was conducted. Box plots depict the Jaccard dissimilarity distance (0 = samples are identical, 1 = samples are disjoint) between‐subjects and within‐subjects. (B) Comparison of the skin virome across subjects over time. (C) Comparison of the virome of the four types of objects across subjects. Statistical comparisons were performed using Wilcoxon rank sum tests. *p < 0.05, **p < 0.01.

Based on previous findings (Figures 4 and 5), 15 virus families were persistently and consistently found across both anatomical locations of subjects and four types of objects (Orthoherpesviridae, Baculoviridae, Mimiviridae, Herelleviridae, Schizomimiviridae, Polyomaviridae, Poxviridae, Suoliviridae, Straboviridae, Adenoviridae, Phycodnaviridae, Rhabdoviridae, Polydnaviriformidae, Papillomaviridae, Salasmaviridae). Consequently, we evaluated 54 viral species belonging to these 15 viral families. We plotted the frequency of occurrence of selected stable viruses across the skin and objects in the subjects at various time points as a heatmap (Figure 6A). We then performed a statistical analysis of corresponding viral markers (Figure 6B,C). A Jaccard dissimilarity matrix (0 = identical, 1 = separate) was generated to compare within‐ and between‐subject changes in the presence and absence of viral markers. Statistical comparisons for between‐subjects (comparison with all other subject samples) and within‐subjects were performed using the Wilcoxon rank sum test. Figure 6B shows a comparison of within‐subject samples over time, with significant differences observed between variations within‐ and between‐subjects for skin (*p* < 0.01). Figure 6C shows a comparison of within‐subject samples across four types of objects, with significant differences observed for inter‐object variations within one subject and between different samples (*p* < 0.05).

## 4 Discussion

Among potential alternative biological data usable as forensic tools, microbial communities can be cited as an example (Zhang et al. 2023). These communities exhibit significant diversity unique to each individual, with specific microbial populations potentially persisting over extended periods (Fierer et al. 2010). However, viruses, which are closely associated with bacteria and can provide a source of abundant genetic diversity, are still not well understood yet.

With their host specificity and high mutation rates, viruses can form unique molecular characteristics for each individual and respond sensitively to lifestyle factors or environmental exposures (Bohannan and Lenski 1997; Rodriguez‐Brito et al. 2010; Modi et al. 2013). These features suggest that virome data have the potential to complement conventional DNA profiling or microbiome analyses by simultaneously providing both individual discriminatory power and contextual information. In fact, individual uniqueness and temporal stability have been repeatedly observed in the human virome, including the skin, supporting its potential for forensic application (Hannigan et al. 2015; Shkoporov et al. 2019). Furthermore, the recent decline in sequencing costs, advances in computational resources, and the expansion of bioinformatic tools have accelerated metagenome‐based virus research, making its implementation increasingly feasible even in standard molecular biology laboratories (Chiu and Miller 2019). In short, the virome represents a promising source of information that can complement existing tools while enabling new levels of forensic interpretation.

Here, we described the diversity and stability present in the human skin virome from Korean using temporal information from three time points over 3 months across anatomical locations (forehead, left hand, and right hand). Furthermore, we evaluated the potential sharing and stability of the skin virome with four specific object surfaces. In summary, this study confirmed the feasibility of performing virus‐based analyses for forensic purposes such as individual identification and linking individuals with objects.

We identified 2400 viral contigs in the skin virus metagenome. Among these contigs identified in this study, those belonging to tailed bacteriophages (Caudoviricetes class within the Duplodnaviria realm) showed an overwhelming dominance (76%), followed by a significant distribution of ssDNA from the Monodnaviria realm (15%) (Figure 1). Previous studies have reported that skin virome mainly consists of Caudoviricetes bacteriophages (Schwede 2003; Oh et al. 2016). Additionally, the Monodnaviria realm, including Papillomaviridae and Polyomaviridae families, has been repeatedly identified as part of the healthy human virome (Koonin et al. 2021). Therefore, our results are consistent with the previous report. However, the current viral reference databases contain a disproportionate number of Caudoviricetes genomes (Graham et al. 2023), whereas certain viral lineages remain underrepresented due to difficulties in isolation and sequencing. Therefore, the observed predominance may reflect database limitations rather than true biological dominance. In fact, approximately 59.31% of contigs in this study remained unclassified, indicating that existing databases do not yet adequately encompass viral diversity. Thus, virome‐related studies, including ours, may be influenced by annotation bias, and their results should be interpreted with caution. To fully understand the human skin virome, further research focusing on virus identification, isolation, annotation, and comparative genomics is necessary.

To assess the forensic applicability of the virome for individual identification, we analyzed the abundance of viruses across anatomical locations and time points (Figure S2). The relative abundance of these contigs varied across subjects. However, there was minimal variation in the overall composition of the virome across anatomical locations at the same time points of each subject. In addition, the relative abundance of the virome within subjects remained consistent over the three sampling time points spanning 3 months. In essence, we found that while the relative abundance of the most abundant annotated contigs varied between subjects, there were no significant fluctuations over time or across anatomical locations within individuals (Figure S2). Statistical evaluation confirmed that there was no significant difference in the diversity (α‐diversity) of samples within participants (*p* = 0.620), although there was a significant difference in the diversity (β‐diversity) of samples between subjects (*p* = 0.001) (Figure S3). This suggests the potential use of virus markers for forensic purposes.

We selected four types of objects to assess object‐to‐skin transferability. We differentiated objects handled by subjects in their daily lives (cell phones, door handles) from those temporarily touched (fabric, plastic). Objects encountered in daily life were selected considering that they are commonly possessed by all subjects. Conversely, objects that are in temporary contact were selected concerning previous studies (Lee et al. 2016; Kodama et al. 2019; Neckovic et al. 2020), taking into account the following: (1) transferability of microbial communities has been observed, (2) they can be sterilized in advance, and (3) they can be collected as forensic evidence. The relative abundance was visualized based on the top 10 most abundant viral families in hosts and objects (Figure 2). Despite differences in object type and contact time, viral abundance among objects was similar to that of the host. This might be because the transfer of bacteria from skin to objects has reached a plateau after a certain number of contacts without accumulating more bacteria as contact increases (Wang et al. 2022). However, research on the relationship between the amount of bacterial and viral transmissibility or surface characteristics is still in its early stages (Hoisington et al. 2023). It remains unclear whether viral transmissibility is due to subject behavior (e.g., frequent handling of cell phones and door handles, having many visitors, frequent cleaning) or if specific viruses might move more prominently. Nonetheless, to the best of our knowledge, this is the first study to demonstrate the possibility of associating viral traces of human contact with objects and places (Figures 2 and 3).

To evaluate the possibility of individual identification based on stability and transmissibility as viral markers, we identified viral families that were reliably detected at sampling points (2 out of 3 times) (Figure 4) and objects (2 out of 4 total) (Figure 5). Consequently, we identified 15 stable viral families commonly observed on skin and objects (Figures 4 and 5). Viruses belonging to the previously mentioned Caudoviricetes and Papovaviricetes were mainly observed. Viruses belonging to the Varidnaviria realm were also observed. Varidnaviria is a relatively small component of the healthy human virome (Koonin et al. 2021). In addition, Baculoviridae and Polydnaviriformidae were observed (Figures 4 and 5). Baculoviridae was previously identified as a stable virus in skin virome studies aimed at individual identification (Graham et al. 2022). In summary, we identified 54 potential virus markers belonging to 15 viral families (Figure 6A). Significant differences were observed between variations within and between subjects for anatomical locations (*p* < 0.01) (Figure 6B). Additionally, significant differences were observed between inter‐object variations within one subject and between different samples (*p* < 0.05) (Figure 6C). This suggests the potential of using viral markers that are stable within individuals to align with the individual from whom they are presumed to have originated through objects.

Recent studies suggest the practical potential of the skin virome for personal identification and contact tracing. Hannigan et al. reported that the skin virome maintains a stable, individual‐specific composition over several months (Hannigan et al. 2015), and Graham et al. further highlighted its forensic relevance by showing both distinct individual specificity and temporal consistency of the skin virome (Graham et al. 2022). These findings indicate that the virome may serve as a complementary or alternative tool for human identification, particularly in cases where traditional STR‐based DNA analysis is not feasible or yields limited information. In addition, bacterial communities recovered from frequently used personal items have shown high concordance with the microbiome profiles of their users, supporting the feasibility of microbiome‐based forensic markers (Fierer et al. 2010). Taken together, these studies suggest that the virome holds substantial promise as a novel type of forensic evidence that can supplement existing DNA‐based approaches.

However, this scientific potential is accompanied by important ethical and legal considerations. Even though virome or microbiome data are not directly linked to human genetic information, they can nonetheless reflect sensitive attributes such as health status, lifestyle patterns, and medication history, raising concerns about unintended inference and associated privacy risks (Rhodes 2016; García et al. 2020). Consequently, the development and application of virome‐based forensic databases must be guided by multilayered ethical discussions that incorporate explicit, purpose‐limited consent procedures, strict oversight of secondary data use, and appropriate legal safeguards. Addressing these issues will require coordinated engagement across forensic, scientific, and legal communities. Once these ethical, institutional, and technical standards are established, the virome has the potential to serve as a valuable independent or complementary marker for forensic investigations.

The limitations of this study are as follows. First, this study involved only eight subjects and was conducted over a 6‐month period, which may introduce population‐specific biases and limit statistical power. In particular, the small sample size posed limitations in applying multivariate modeling. Moreover, as a previous study has tracked the skin virome for up to 6 months (Graham et al. 2022), longer‐term observations are warranted. In future work, we plan to expand cohort diversity, considering factors such as age, skin health, and environmental exposures, and to conduct a follow‐up study with a larger number of Korean subjects over at least 1 year. Through these efforts, we aim to apply advanced statistical methods to more robustly verify the stability and transferability of the virome and to enhance the generalizability and reliability of its application in forensic science. Second, questionnaire‐based data were not included as covariates in the statistical analysis of the virome. Subjects completed questionnaires on intrinsic and extrinsic factors potentially affecting skin viral clusters (Figure S1). These confirmed a minimum interval of 2 h since the last handwashing and no antibiotic use within 30 days prior to sampling. However, these factors were treated only as contextual information rather than as adjusted covariates. Future research with larger sample sizes should incorporate such behavioral data as covariates to improve the accuracy and robustness of virome analyses. Third, virome stability and transmissibility were evaluated only under controlled conditions. In practice, forensic samples are often degraded or contaminated by environmental factors (e.g., temperature, humidity), which may affect analytical results (Lindahl 1993; Bonfigli et al. 2023). Therefore, future studies should validate findings using mock forensic samples under diverse environmental conditions and assess their applicability in real‐world forensic casework.

## 5 Conclusions

We identified 54 viral markers that demonstrate stability and transmissibility across anatomical locations, associations with objects, and individual characterization from skin metagenomic samples of Koreans. This research supports the potential role of the virome in forensic applications and will aid future studies in viral forensics. However, several challenges persist in the field of virome research. For example, the scarcity and bias of reference databases, along with the lack of standardized sequence clustering methods, continue to constrain progress (Chang et al. 2024). Furthermore, the virome may reveal personal health information such as age, sex, and disease status, which could raise privacy and ethical concerns if applied as a forensic identifier. Addressing these challenges through future research and institutional deliberation will be essential for advancing the practical feasibility of virome applications. In summary, the development of viral metagenomics provides a novel approach to forensic investigation and contributes to the advancement of forensic science as an alternative form of biological evidence.

## Author Contributions

**Min‐Jeong Kim:** conceptualization, formal analysis, investigation, methodology, software, validation, visualization, writing – original draft. **Ji‐Ho Park:** conceptualization, formal analysis, investigation, methodology, software, validation, visualization, writing – original draft. **Yong‐Bin Eom:** conceptualization, funding acquisition, methodology, project administration, resources, supervision, validation, writing – original draft, writing – review and editing.

## Ethics Statement

This study was approved by the Institutional Review Board of Soonchunhyang University (IRB approval number: 1040875‐202306‐BR‐068). Written informed consent was obtained from all participants.

## Conflicts of Interest

The authors declare no conflicts of interest.

## Supporting information


**Figure S1:** A questionnaire designed to investigate factors that might influence skin virome. All subjects were asked to complete this questionnaire before each sampling. **Figure S2:** Relative abundance of the top ten most abundant viruses identified at each time point in each subject's anatomical location. **Figure S3:** The diversity of the skin virome.

## Data Availability

The data that support the findings of this study are openly available in NCBI databases at https://www.ncbi.nlm.nih.gov/sra, reference number PRJNA1073697. The sequence data have been submitted to the NCBI databases under accession number PRJNA1073697. Addresses are as follows: https://www.ncbi.nlm.nih.gov/sra.
