# Supplementary material for: The Transmissibility of the Human Skin Virome: Potential Forensic Implications
Source: Microbiologyopen. 2025 Dec 8;14(6):e70197. doi: 10.1002/mbo3.70197 (PMC12683279; doi:10.1002/mbo3.70197)
Supplement: Supplementary file 1 — Figure S1: A questionnaire designed to investigate factors that might influence skin virome. All subjects were asked to complete this questionnaire before each sampling. Figure S2: Relative abundance of the top ten most abundant viruses identified at each time point in each subject's anatomical location. Figure S3: The diversity of the skin virome. [file MBO3-14-e70197-s001.pdf]

Questionnaire

[Related to Skin Virome Research]

Date:

Subject Number:

1. Please check the box that applies to you.

1)	Have you traveled to a place you don't usually visit in your daily life in the past 7 days?	Yes<br>☐ (Is it within the country or abroad?)		No<br>☐
2)	Did you use cosmetics? If so, what type of cosmetics did you use? (e.g., sunscreen, foundation, etc.)	Yes<br>☐ (Cosmetic types :)		No<br>☐
3)	Approximately how long has it been since you last washed your hair?	Washing<br>☐ (Time and duration :)		Do not know<br>☐
4)	Approximately how long has it been since you last washed your hands?	Washing<br>☐ (Time and duration :)		Do not know<br>☐
5)	Do you use hand lotion/cream? If so, approximately how long has it been since you last used it?	Used<br>☐ (Time and duration :)	Not used<br>☐	Do not know<br>☐
6)	Do you use gel -type hand sanitizer? If so, approximately how long has it been since you last used it?	Used<br>☐ (Time and duration :)	Not used<br>☐	Do not know<br>☐
7)	Have you visited a swimming pool in the last 30 days? If so, approximately how long has it been since your visit?	Yes<br>☐ (Time and duration :)	No<br>☐	Do not know<br>☐
8)	Have you taken antibiotics in the last 30 days? If so, approximately how long has it been since you took them?	Yes<br>☐ (Time and duration :)	No<br>☐	Do not know<br>☐

2. Is there any other information related to the skin that you think is relevant and would like to share?

(Feel free to provide any additional information in your own words.)

Figure S1 A questionnaire designed to investigate factors that might influence skin virome. All subjects were asked to complete this questionnaire before each sampling.

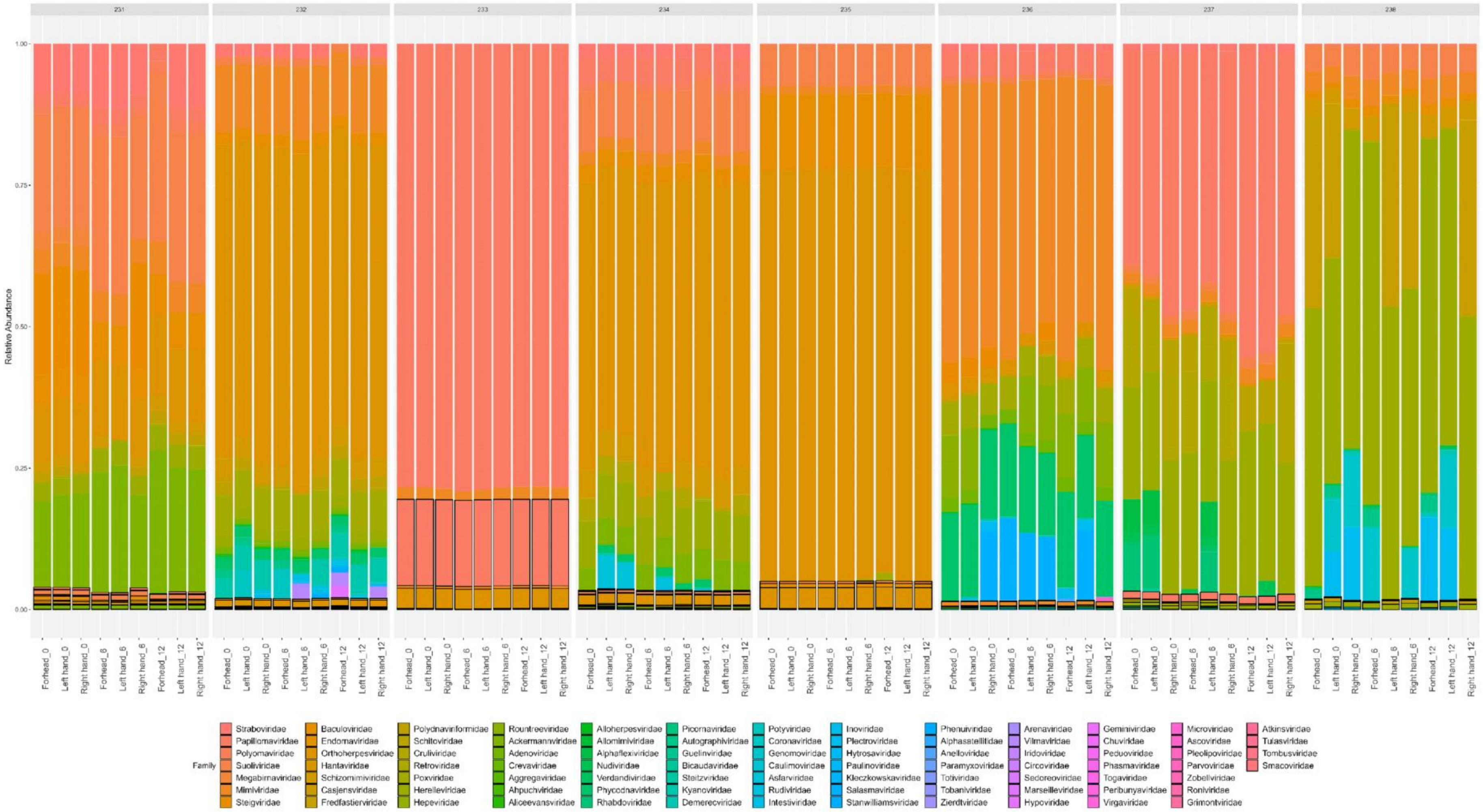

Figure S2 Relative abundance of the top ten most abundant viruses identified at each time point in each subject's anatomical location. The subject's number is indicated at the top of the bar. Each bar represents the subject's anatomical location and sampling time point (location: forehead, left hand, right hand, and time point (week): 0, 6, 12). Contigs that could not be taxonomically classified at the family level using the current database were not included.

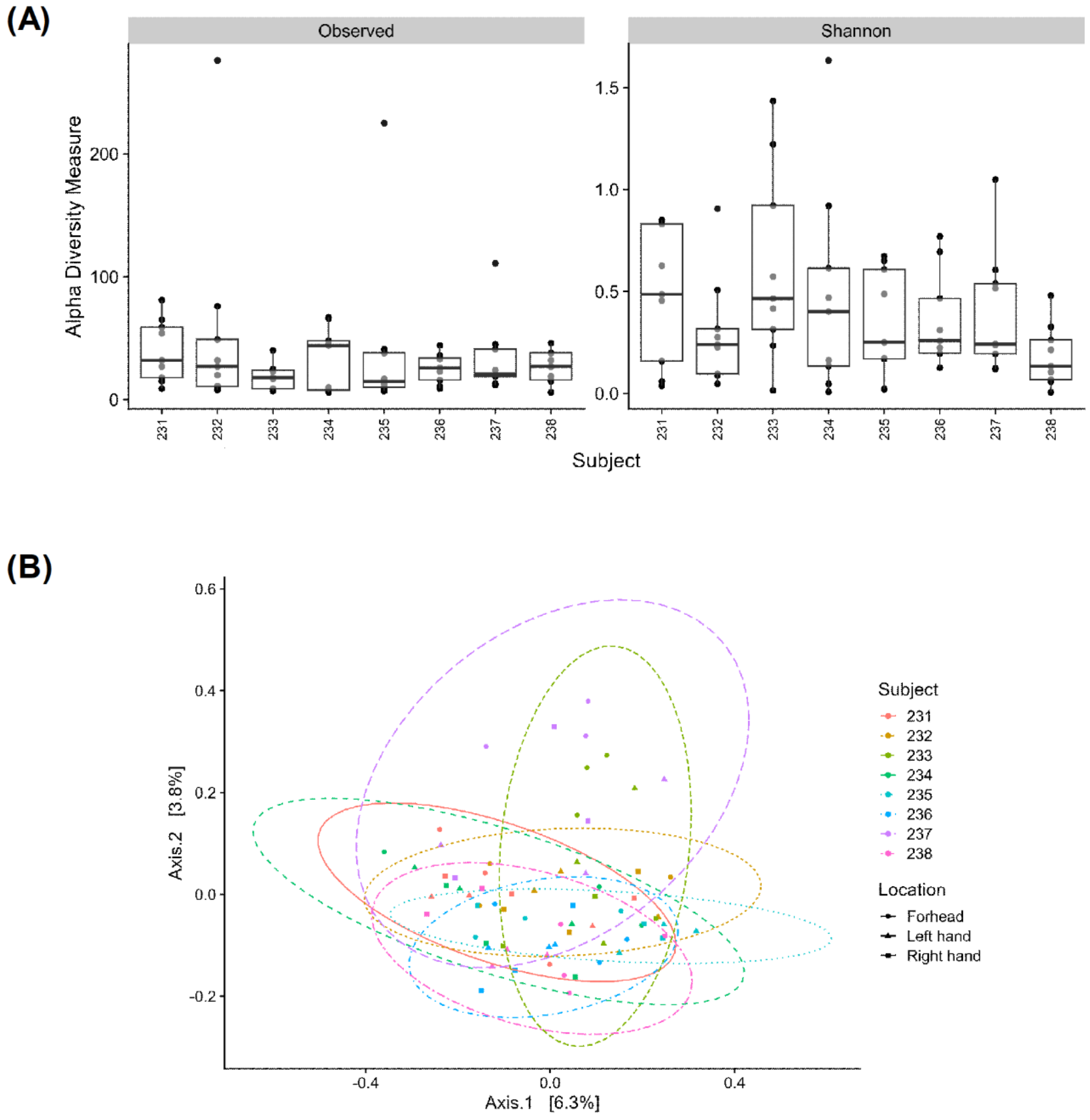

Figure S3 The diversity of the skin virome. (A) Box plot comparing Shannon diversity measurement values for skin samples of subjects. ANOVA for subject anatomical locations showed no significance ($p = 0.620$). (B) PCoA plots of the Jaccard dissimilarity distance for β-diversity assessment. In PERMANOVA, clustering by subject according to skin virome showed significance ($R^2 = 0.16$, $p = 0.001$).
